# Alternative mechanisms of miR-34a regulation in cancer

**DOI:** 10.1038/cddis.2017.495

**Published:** 2017-10-12

**Authors:** Eva Slabáková, Zoran Culig, Ján Remšík, Karel Souček

**Affiliations:** 1 Department of Cytokinetics, Institute of Biophysics of the Czech Academy of Sciences, Brno, Czech Republic; 2 Center of Biomolecular and Cellular Engineering, International Clinical Research Center, St. Anne’s University Hospital Brno, Brno, Czech Republic; 3 Division of Experimental Urology, Department of Urology, Medical University of Innsbruck, Innsbruck, Austria; 4 Department of Experimental Biology, Faculty of Science, Masaryk University, Brno, Czech Republic

## Abstract

MicroRNA miR-34a is recognized as a master regulator of tumor suppression. The strategy of miR-34a replacement has been investigated in clinical trials as the first attempt of miRNA application in cancer treatment. However, emerging outcomes promote the re-evaluation of existing knowledge and urge the need for better understanding the complex biological role of miR-34a. The targets of miR-34a encompass numerous regulators of cancer cell proliferation, survival and resistance to therapy. MiR-34a expression is transcriptionally controlled by p53, a crucial tumor suppressor pathway, often disrupted in cancer. Moreover, miR-34a abundance is fine-tuned by context-dependent feedback loops. The function and effects of exogenously delivered or re-expressed miR-34a on the background of defective p53 therefore remain prominent issues in miR-34a based therapy. In this work, we review p53-independent mechanisms regulating the expression of miR-34a. Aside from molecules directly interacting with MIR34A promoter, processes affecting epigenetic regulation and miRNA maturation are discussed. Multiple mechanisms operate in the context of cancer-associated phenomena, such as aberrant oncogene signaling, EMT or inflammation. Since p53-dependent tumor-suppressive mechanisms are disturbed in a substantial proportion of malignancies, we summarize the effects of miR-34a modulation in cell and animal models in the clinically relevant context of disrupted or insufficient p53 function.

## Facts

- MiR-34a expression is lost or decreased in many cancers
- Re-expression of miR-34a has been investigated in clinical trials as potential treatment of advanced cancers
- MiR-34a contributes to tumor suppression by repressing over 700 transcripts implicated in cellular proliferation, survival and plasticity
- MiR-34a expression is governed by p53, but can be regulated by multiple p53-independent mechanisms

## Open Questions

- Can miR-34a exert any pro-tumorigenic effects in a specific context?
- How can miR-34a modulation affect human immune response and the condition of vital organs? What outcomes of systemic miR-34a delivery can be expected in p53-defective cancer cells and in the microenvironment of p53 wild-type normal cells?
- Is optimization of the delivery method sufficient to avoid potential adverse effects of miR-34a application?

MicroRNAs (miRNA) are evolutionary highly conserved non-coding RNA molecules, exerting essential functions in a wide range of physiological processes. In cancer, miRNAs exert both pro- and anti-tumorigenic effects by virtue of miRNA-specific and context-dependent mechanisms.^1^ Consequently, deregulation of miRNA expression was reported in most cancer types and at multiple levels or miRNA expression control.^2, 3^ The strategy of replacing downregulated miR-34a by intravenous liposome-based delivery has been investigated in phase I clinical trials for advanced stages of multiple solid and hematological malignancies.^4, 5^ The study was recently terminated and reported immune-related adverse effects in several individuals, implicating an urgent need to improve the tolerability of miR-34a-based therapy. Better understanding of the complex biological function of miR-34a in both normal and cancer cells is indispensable for this achievement.

miR-34a was selected as a candidate tumor suppressor miRNA based on its frequent deregulation in cancer tissues^6, 7, 8^ and its ability to regulate the expression of multiple targets implicated in tumorigenesis and cancer progression, such as MYC, MET, CDK4/6, NOTCH1, BCL2, CD44 and many other molecules.^9, 10^ Broad target specificity of miRNAs, resulting from their short binding motifs in target gene sequences, can be advantageous for simultaneous targeting of multiple tumor-promoting transcripts, but at the same time poses a risk of potential adverse effects. Thorough understanding of associated signaling pathways both upstream and downstream of miR-34a is therefore a prerequisite for successful therapeutic application.

Expression of miRNA transcripts is driven from promoter regions that accommodate binding sites of canonical transcription factors. Another level of regulation of miRNA expression by feedback loops was described for multiple miRNA-families including miR-34a.^11, 12, 13^ Transcription of miR-34a is regulated dominantly by the crucial tumor suppressor p53, by means of binding to multiple canonical p53 binding sites in regions proximal to the MIR34A promoter.^14, 15, 16^ Importantly, miR-34a is detected likewise in tissues and cells with p53-mutation or deletion, implicating the existence of p53-independent mechanisms of miR-34a expression.^17, 18^

Mutation or inactivation of the tumor suppressor p53 occurs in a high proportion of tumors,^19^ affecting cell proliferation, survival and sensitivity to chemotherapy. This is often associated with downregulation of miR-34a expression in both hematologic and solid malignancies.^18, 20, 21, 22, 23, 24^ Due to the implication of miR-34a in multiple feedback loops, which can be strongly affected by the therapeutic dose of miR-34a mimic, it is important to consider potential effects of miRNA-based anti-cancer therapy in the context of disrupted or insufficient p53 function. Important questions arising from this presumption are: (1) Which p53-independent mechanisms can affect miR-34a expression? (2) What may be the consequences of miR-34a modulation in cancers harboring defects in p53 function?

## miR-34 family, biogenesis, targets and expression

Of the 3 members of miR-34 family, miR-34a is ubiquitously expressed in normal human tissues, while expression of miR-34b/c is characterized by tissue specificity to the testicles, fallopian tubes, lungs or brain.^7^ In human genome, miR-34a is encoded on chromosome 1p36, while miR-34b and miR-34c are expressed from one common transcript of chromosome 11q23.^10, 25^ Similarly to the biogenesis of all miRNAs, miR-34a is transcribed as a long hairpin molecule (pri-miRNA), which is subsequently cleaved by an RNase III Drosha to an ~70-nucleotide long stem-loop precursor (pre-miRNA). Following nuclear export, the pre-miRNA is further cleaved by an RNase III Dicer into 22-nucleotide long mature strands, which are incorporated into RNA-induced silencing complex (RISC). This RNA/protein complex mediates downregulation of target transcripts by mRNA degradation or inhibition of translation.^9, 10^ In case of miR-34a, experiments with synthetic pre-miRNAs revealed that incorporation of both 5p and 3p mature strands into RISC enables specific regulation of different targets.^26^

MiR-34a is considered to act as a tumor suppressor miRNA, since of the 700 to date experimentally validated miR-34a targets,^27^ many genes are implicated in the control of cellular proliferation (that is, cyclins, cyclin-dependent kinases, MYCN, NOTCH1, MDMX), apoptosis (BCL2, SIRT1 and BIRC5), senescence (E2F3), cancer stem-like cell phenotype (CD44, NANOG and SOX2), motility (SNAI1, MET and AXIN2) or immune evasion (PD-L1, DGK*ζ*). MiR-34a therefore exerts wide-range effects on cancer progression and metastasis (for overview see references 9, 10, 28, 29 and an updated database of validated miRNA targets^27^).

In accordance with general downregulation of miRNA expression in malignancies,^30^ downregulation of miR-34a expression was reported in multiple types of cancer.^6, 7, 8, 25, 31^ Still, well designed cohort studies are required to establish miR-34a as a prognostic factor.^32^ Concomitantly, the genomic locus 1p36 encoding miR-34a transcript is lost in certain tumors,^33^ proposing one of possible mechanisms of miR-34a loss in neuroblastoma.^34, 35^

## p53-dependent regulation of miR-34a expression and function

In physiological conditions, expression of miR-34a is transcriptionally regulated by the key tumor suppressor p53. The function of p53 in the prevention of uncontrolled proliferation of cells with damaged DNA predisposes p53 to be one of the most frequently inactivated proteins in human cancer. Activation of p53 results either in cell cycle arrest enabling repair of minor damage, induction of replicative senescence, or apoptosis. Mechanisms of evasion from p53-mediated tumor suppression encompass selection of cancer cells harboring p53 mutations (nonsense or missense, eventually accompanied by a gain of function) or allelic loss, as well as inactivation by viral or cellular proteins.^19, 36^

**Figure 1:**
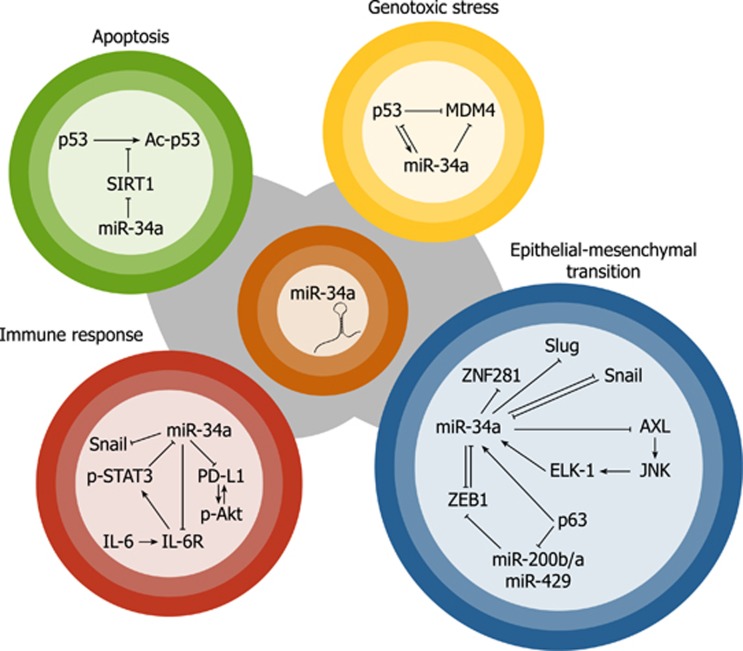
Feedback regulation of miR-34a. Upon DNA damage, miR-34a transcription is induced by p53. miR-34a targets p53 directly by interaction with its transcript and indirectly through targeting of p53 inhibitor MDMX (yellow). MiR-34a-mediated inhibition of p53 deacetylase SIRT1 is implicated in apoptosis (green). Feedback inhibition between miR-34a, IL-6R and P-STAT3 is implicated in inflammation, while regulation of PD-L1 – P-Akt axis is important for immune surveillance (red). In EMT, miR-34a is implicated in multiple feedbacks encompassing EMT regulators Snail, ZEB1 or AXL (blue)

An evolutionary conserved p53-binding site was identified upstream of the miR-34a transcript.^14, 15, 37^ Additional interaction between p53 and an intronic region of miR-34a was shown to be enhanced by genotoxic stress.^37^ On the other hand, several feedback loops implicate miR-34a in the regulation of p53 (Figure 1). For example, TP53 mRNA was shown to be targeted by miR-34a through non-canonical response elements in 5′UTR and the coding sequence.^38^ MDMX, an inhibitor of p53 transactivation, is a direct target repressed by miR-34a.^39, 40^ From epigenetic mechanisms, miR-34a represses the histone deacetylases SIRT1 and HDAC1, thereby enhancing the ability of p53 to transactivate its target genes.^13, 41^

Induction of miR-34a expression by genotoxic stress is strongly p53-dependent,^6, 15, 37, 42, 43, 44^ with only one reported exception in bladder cancer cells.^45^ However, a correlation between basal level of miR-34a and p53 status was demonstrated only in a proportion of experimental models.^13, 14, 17, 46, 47, 48^ A lack of significant correlation between p53 mutational status and miR-34a expression was observed in a set of lung cancer patients,^42^ pancreatic cancer and CSC-like cells^49^ and colorectal cancer,^50^ suggesting either that p53 transcriptional activity rather than mutation status is essential for miR-34a expression, or that the basal level of miR-34a expression can be maintained by p53-independent mechanisms as well.

On the other hand, members or the miR-34 family were proposed to be key mediators of p53 tumor suppressor function after DNA damage. In most conditions, miR-34a overexpression inhibited cell proliferation or induced a senescent phenotype,^6, 15, 37, 42, 43^ while induction of apoptosis after miR-34a overexpression was observed only in certain experimental models.^6, 14, 15, 37^ The level of miR-34a expression was proposed as a mechanism responsible for cell-fate decision after p53 induction.^51^ Importantly, miR-34a inhibition consistently desensitized cells to apoptosis induced by genotoxic stress.^15, 28, 42, 45, 52^

Nevertheless, recent reports show that p53 can exert its function even in the absence of miR-34a. It was demonstrated that the response to genotoxic stress is intact in miR-34 KO cells and animals, but this effect may result from redundancy between miR-34 and miR-449 families, sharing the same seed.^17, 38^ This hypothesis is supported by facts that miR-34/449 double KO mice exhibited postnatal mortality, infertility and strong respiratory dysfunction caused by defective mucociliary clearance,^53^ and that deletion of miR-34b/c cluster induces expression of miR-449 family, and vice versa.^54^ In a Kras-induced mouse lung cancer model, miR-34a deficiency alone does not exhibit a strong oncogenic effect. However, miR-34a deficiency strongly promotes tumorigenesis when p53 is haploinsufficient, suggesting that the defective p53-miR-34 feedback loop can enhance oncogenesis in a specific context.^40^ Consistently, prostate epithelium-specific inactivation of miR-34 and p53 leads to expansion of the prostate stem cell compartment and development of early invasive adenocarcinomas and high-grade prostatic intraepithelial neoplasia through enhanced MET signaling.^55^

## p53-independent mechanisms of miR-34a regulation

**Figure 2:**
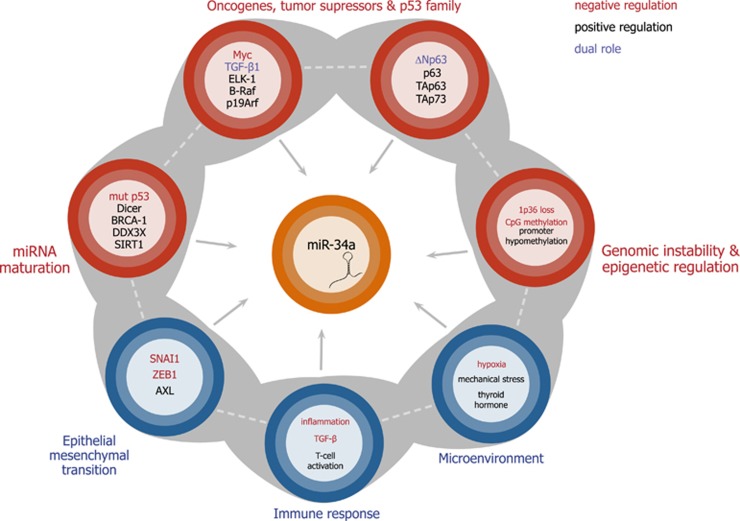
Reported mechanisms of p53-independent miR-34a regulation in different biological contexts. Intrinsic/intracellular mechanisms are represented in red, extrinsic mechanisms triggered by intercellular or microenvironmental effects are depicted in blue. Notably, certain molecules are characterized by context-dependent roles in the regulation of miR-34a

Multiple experimental observations suggest that besides p53-driven miR-34a expression, miR-34a levels can be regulated in a p53-independent manner.^42, 49^ Mechanisms responsible for p53-independent regulation can either operate simultaneously with p53-dependent control, or establish dominance in case of disrupted p53 function. From the multitude of miR-34a influencing factors, some can be classified as extrinsic (triggered by activation of signaling pathways by external stimuli, such as in the case of factors originating from cell microenvironment, or epithelial–mesenchymal transition (EMT)-associated changes), while factors classified as intrinsic act at the level of intracellular signaling pathways, epigenetic regulation or affect the general process of miRNA biogenesis (Figure 2). While certain miR-34a regulating mechanisms were described in physiologic conditions, other are associated with disease-related processes such as inflammation or oncogene signaling.

### Regulation of miR-34a expression in the process of miRNA maturation

miRNA maturation involves two subsequent RNA cleavage steps, mediated by RNase III enzymes Drosha and Dicer, respectively. The assembly, activity and target recognition by the Drosha complex require cooperation with DGCR8 and auxiliary factors, such as the DEAD-box RNA helicases p68 (DDX5) and p72/p82 (DDX17).^56^ During the second cleavage step, Dicer is associated with auxiliary proteins, such as TAR RNA binding protein and kinase R–activating protein to increase its stability and processing activity.^57^

The expression and assembly of molecules involved in the miRNA processing machinery was shown to be affected by transcription factors primarily associated with other biological processes, such as components of p53, TGF-*β* or Hippo signaling pathways.^58, 59, 60, 61^ The maturation of several miRNAs, but not miR-34a, was enhanced by p53 activation after genotoxic treatment.^61^ Importantly, transcriptionally inactive p53 mutants interfered with a functional assembly between Drosha complex and RNA helicases DDX5/DDX17, leading to attenuation of miRNA processing activity.^61, 62^ TAp63, except from directly affecting MIR34A transcription by binding to its promoter, can also enhance miR-34a processing by transcriptional activation of Dicer.^60^

A specific effect on miR-34a maturation was observed after BRCA1 overexpression, which accelerated the processing of miRNA primary transcripts, thereby increasing the expression of both precursor and mature forms of miR-34a. Despite physical association of BRCA1 with p53, the mechanism was reported in cell lines with disturbed p53 function resulting from immortalization or p53 mutation.^63^ Modulation of SIRT1 activity influenced miR-34a maturation in keratinocytes with both normal and reduced p53 function.^64^

Furthermore, miR-34a was identified as one of six miRNAs whose expression was affected by knock-down of RNA helicase DDX3X, exerting key roles in cancer development. DDX3X interacts with Drosha/DGCR8 complex, facilitates pri-miRNA binding and promotes miR-34a maturation.^65^ In cells with undisturbed RNA processing machinery, cell type-specific differences in miR-34a abundance were attributed to unequal processing of its pri-miRNA, resulting in differences in cell fate after p53 activation.^51^

### Regulation of miR-34a expression by epigenetic mechanisms

The phenomenon of CpG methylation, resulting in inactivation of the surrounding chromatin due to recruitment of histone deacetylases, is important in the maintenance of cell specific expression patterns and is stably inherited to the next cell generation.^66, 67^ Both miR-34 transcripts contain a CpG island in their promoter region.^68^ CpG methylation of the MIR34A promoter was observed in almost 80% of primary prostate carcinomas and a variable proportion of other tumors. In cancer cell lines, the frequencies of MIR34A methylation varied from 13% in colon cancer to 43% in melanomas.^69^ Contrarily, in formalin-fixed, paraffin-embedded tumor samples, MIR34A methylation was the most frequently detected in colorectal cancer (74%). Data from other tumor types also suggest that MIR34A promoter methylation occurs more frequently *in vivo* than *in vitro*.^70^ Importantly, MIR34A promoter methylation correlated with distant metastases in colon cancer patients,^71^ underscoring the clinical importance of miR-34a implication in cellular plasticity.^12^

Contrarily, promoter hypomethylation was responsible for induced miR-34a expression in alcoholic liver injury. In normal human hepatocytes and cholangiocytes, induction of miR-34a enhanced cell survival and migration, which in the context of liver cells may be associated with tissue remodeling and regeneration.^72^ Hypomethylation of an alternative promoter region upstream from MIR34A coding sequence was observed in chronic lymphocytic leukemia compared to healthy B cells, which was accompanied by increased miR-34a expression in malignancy.^68^

Mechanistically, a lack of miR-34a induction in cells with wild-type (wt) p53 after DNA damage was attributed to chromatin methylation in IGR-39 melanoma cells.^69^ Restoration of miR-34a expression by chromatin modulators affected expression of miR-34a targets, cell survival and EMT phenotype in pancreatic cancer cell lines.^49^ Analysis of MIR34A methylation in a wide cohort of primary colorectal cancer samples pointed out a statistically significant correlation of MIR34A methylation and the absence of p53 mutation (evaluated by immunohistochemical score of p53), indicating that the loss of the miR-34a-mediated tumor suppressor function may substitute for loss of the p53 response by p53 mutations in colorectal cancer.^70^ Similarly, low incidence of p53 mutation together with epigenetic mechanism of miR-34a silencing was reported in diffuse large B-cell lymphoma.^73^ On the other hand, in the context of disrupted p53, reintroduction of miR-34a induced a senescent phenotype in TP53-null PC3 cells and p53 mutated (R248W) MIA PaCa-2 cells, characterized by a moderate and high level of MIR34A promoter methylation, respectively.^69^ These findings suggest that the therapeutic strategy of miR-34a reintroduction might be beneficial for patients with epigenetically downregulated miR-34a, regardless of the p53 status in tumor cells.

### Regulation of miR-34a expression by members of the p53/p63/p73 family of transcription factors

Homologs of the p53 tumor suppressor, p73 and p63, share a high degree of structural similarity and can bind and activate transcription from the majority of p53-responsive promoters. Transcription from alternative promoters and splicing events give rise to full length, transactivation domain containing (TA) and truncated (ΔN) isoforms of p63 and p73, with distinct functions and physiological roles.^74^

Direct binding to the p53 consensus DNA-binding sites in MIR34A promoter was demonstrated for both p63 and p73.^60, 75, 76, 77^ The TAp73-miR-34a axis represents a positive regulation, restricted to miR-34a and implicated in neuronal physiology and pathology.^75^ Similarly, *Trp63* KO MEFs exhibit decreased expression of miR-34a and ectopic expression of the ΔNp63*β* isoform induces miR-34a.^76^ On the contrary, ΔNp63 represses miR-34a and miR-34c transcription in murine epidermal cells and enables cell cycle progression in a p53-independent manner,^77^ suggesting context and isoform-specific effects of p63 on miR-34a expression. Furthermore, TAp63 expression is stimulated by a miR-34a target Oct-4, which contributes to oncogenic transformation in the process of pluripotency induction, indicating the importance of miR-34a regulation by p63 in carcinogenesis.^78^

### Regulation of miR-34a in the context of immune response

Chronic inflammation has long been associated with tumor initiation, progression and invasion.^79^ Secreted molecules implicated in inflammation such as TNF-*α*, IL-6 or LPS were described as potent inducers of EMT and key players in cancer progression.^80^ In accordance with its reported anti-tumorigenic function, miR-34a exerts an anti-inflammatory effect by downregulating TNF-*α* and IL-6.^81^ Expression of miR-34a itself can be affected by inflammatory stimuli, as it was found downregulated after LPS stimulation in macrophages^81^ or upregulated by atherosclerosis-inducing oscillatory shear stress in endothelial cells.^82^ MiRNA profiling of wt *versus TP53* KO mice infected by *Corynebacterium parvum* demonstrated that inflammation-associated upregulation of miR-34 family members is largely p53-dependent.^83^ Likewise, dependence on intact p53 function was demonstrated for induction of miR-34a by direct binding of immune response-associated transcription factor NF-*к*B to MIR34A promoter.^84^

A p53-independent mechanism of miR-34a regulation in inflammation is exemplified by direct repression of MIR34A gene *via* a conserved STAT3-binding site in the first intron during IL-6–induced EMT and invasion in colorectal cancer cells harboring p53 mutation^11^ (Figure 2). Nevertheless, p53-dependent expression of miR-34a was crucial for suppression of tumor progression by inhibiting the IL-6R/STAT3/miR-34a feedback loop (Figure 1).^11^

As an upstream regulator of PD-L1, CCL22 and DGK*ζ* expression, miR-34a was identified as an important regulator of immune response in cancer.^31, 85, 86^ MiR-34a overexpression reversed chemotherapeutic agent-induced PD-L1 expression, reduced PD-L1 specific T cell apoptosis and inhibited Treg recruitment in p53-defective models.^31, 86^

### Regulation of miR-34a in the context of EMT

EMT encompasses a series of phenotypic and biochemical changes enabling cell spreading, eventually leading to the formation of metastases or acquisition of chemoresistance.^87^ Multiple external signals, such as cytokine-triggered signaling pathways or more complex forms of cellular or environmental stress (hypoxia, nutrient deprivation, inflammation), converge on the regulation of several nodal EMT-driving transcription factors (Snail, Slug, Twist, ZEB1, ZEB2), responsible for regulation of intracellular and extracellular molecules characteristic for the mesenchymal phenotype.^88^ A significant role of miRNAs in the control of EMT is exemplified by a double-negative feedback loop between ZEB1 and ZEB2 transcription factors and miR-200 family.^89^

A role of miR-34a in the regulation of EMT was described in cancer cell lines, animal models of human cancer as well as in hypoxia-induced EMT in renal tubular epithelial cells, suggesting that miR-34a is a universal regulator of EMT.^8, 11, 12, 76, 90, 91, 92^ Notably, miR-34a expression was shown to be reciprocally controlled by EMT-regulating molecules. Direct binding of Snail or ZEB1 to E-boxes in the promoter regions of both miR-34 family transcripts proved a direct repression of miR-34 transcription by EMT-inducing transcription factors^12^ (Figure 2).

Similarly to the previously reported p53/miR-200/ZEB1/2 axis, both Snail and ZEB1-mediated regulation of miR-34a is subject to regulation by double-negative-feedback loops (Figure 1). Mutual negative regulation between miR-34a and Snail was discovered in the context of p53-dependent mesenchymal–epithelial transition. Besides repression of Snail, miR-34a was shown to negatively regulate Slug, ZEB1, ZNF281 as well as several stemness factors, thereby stabilizing the epithelial phenotype.^12, 93^

In a *Kras/Trp53*-mutation driven mouse model of human non-small cell lung cancer (NSCLC), ZEB1 drove pro-migratory cytoskeletal processes and metastasis by downregulating the expression of miR-34a. In this case, the repression of miR-34a by ZEB1 was indirect, mediated by repression of ΔNp63 by ZEB1. The finding that ΔNp63 serves as a downstream mediator of ZEB1 completes a feedback circuit initiated by p63, which transcriptionally activates the miR-200b/a/429 cluster^94^ and, in turn, directly targets ZEB1 (Figure 1).^76^

Although miR-34a induction was originally observed during p53-dependent restoration of epithelial phenotype, mutual regulation between miR-34a and Snail and prevention of TGF-*β*-induced EMT by miR-34a overexpression was confirmed in models harboring defects in p53 function.^12^ Likewise, the discovery of miR-34a regulation by ZEB1 on the background of identical germline *Trp53* mutation suggests that this mechanism is independent of intact p53 function.^76^ In the context of EMT-associated miR-34a regulation in hypoxia or after thyroid hormone treatment, the model cell line derived from adult human kidney established by transduction with human papilloma virus E6/E7 genes suggests independence of p53 function, although this aspect has not been experimentally addressed.^91, 92^ Nevertheless, direct induction of MIR34A transcription by binding of thyroid hormone receptor to MIR34A promoter region inhibited TGF-*β*1-induced EMT in renal tubular epithelial cells, pointing to another significant mechanism of miR-34a regulation in the context of EMT.^92^

### Regulation of miR-34a in the context of aberrant cancer-related signaling and cancer therapy

The fact that miR-34a expression is often deregulated in cancer suggests a possibility that the expression of miR-34a itself may be regulated by oncogenes or tumor suppressors. In physiological settings, miR-34a expression was proportional to the expression of tumor suppressor p19^Arf^ in a p53-independent manner, with an implication in mouse development through Pdgfr*β* expression.^95^

Aberrant oncogene activation can trigger cellular senescence, an anti-tumor mechanism characterized by permanent proliferative arrest. In the context of senescence, miR-34a was identified as a key mediator of c-Myc and E2F repression, mediating indirect downregulation of an entire set of mitotic genes and telomerase activity.^6, 96, 97^ B-RAF-induced senescence of human fibroblasts was accompanied by p53-independent induction of miR-34a, mediated by an ETS-family transcription factor ELK-1.^96^ ELK-1 was described as a miR-34a regulator also in a feedback regulation of tyrosine kinase AXL, implicated in cancer invasion, EMT and chemoresistance. This feedback loop is activated by AXL overexpression through JNK-mediated ELK-1 activation, and this subsequently leads to an upregulated expression of miR34a that, in turn, downregulates AXL protein expression (Figure 1).^98^

In accordance with context- and target cell-dependent pro- and anti-cancer effect of TGF-*β* signaling, different mechanisms of miR-34a regulation by TGF-*β* were proposed. While computational analysis of microarray data proposes a positive correlation between miR-34a expression and TGF-*β* signaling,^50^ TGF-*β* inhibited miR-34a expression in hepatocellular carcinoma and through miR-34a-CCL22-Treg axis promoted tumor progression and immune escape in p53-deficient cells.^86^ Contrarily, miR-34a induction by TGF-*β* silencing was associated with p53 induction and activation in HeLa cells.^99^

Retinoid therapy, inducing neuroblastoma cell differentiation and growth inhibition, was shown to induce miR-34a levels.^35^ Alternatively to TAp73-mediated induction of miR-34a in retinoid-induced neuroblastoma differentiation,^75^ it is plausible that retinoid-induced downregulation of N-Myc represents another mechanism of miR-34a control.^100^ Gene expression analysis on a panel of breast tumor samples identified a co-regulation between miR-34a and targets of Myc-associated zinc finger protein MAZ,^101^ further corroborating the implication of miR-34a in Myc signaling. In experimental models of Myc-driven neuroblastoma (including a p53-null mouse model), miR-34a was found to be repressed by the oncogene Myc. Although it was demonstrated that Myc binds to the MIR34A promoter,^102^ further studies suggested that epigenetic silencing or chromosomal deletion of the MIR34A genomic locus could be responsible for miR-34a downregulation in neuroblastoma as well.^73^

Epidermal growth factor receptor (EGFR) and hepatocyte growth factor receptor (MET) are tyrosine kinase receptors that have been implicated in the pathogenesis of NSCLC. Combination of miR-34a and let-7 suppressed p53-deficient tumor growth^103^ and demonstrated a synergic anti-proliferative effect with EGFR inhibitor erlotinib, but not other commonly used chemotherapeutics.^104^ MiR-34a also prevented HGF-mediated gefitinib resistance in EGFR mutant lung cancer cells,^105^ suggesting a benefit of adjuvant miRNA-based therapy in p53-deficient NSCLC.

## Effects of miR-34a modulation in the context of deranged p53 function

Modulation of miR-34a levels for therapeutic purpose needs to consider the heterogeneity of target cells and tissues. It was demonstrated that human tumors are complex and non-uniform in terms of expression of many tumor markers^106^ and individual cancer cells can strongly differ even in p53 expression.^107^ Studying the effects of miR-34a in models with disrupted p53 function can therefore help to predict possible effects in tumor cells harboring defective p53, especially regarding the feedback regulation between miR-34a and p53 (Figure 1). Tables 1 and 2 summarize experimental outcomes of miR-34a modulation in the context of insufficient p53 response. Altogether, the effects of miR-34a manipulation may be weaker in p53-disrupted cells than in wild-type p53 background,^46^ but most anti-proliferative and pro-apoptotic effects of miR-34a are maintained regardless of upstream p53 signaling. It is plausible that miR-34a exerts its effects in a complementary and parallel fashion to targets that are directly activated by p53.^42^

## Future outlook

Hundreds of cell- and animal-based studies agree on a tumor-suppressive function of miR-34a and propose restoration of miR-34a expression as a potential therapeutic strategy. The advantage of miR-34a-based therapy is the opportunity to simultaneously repress multiple oncogenic and immune evasion pathways.^4, 21, 48^ While efficiently inhibiting cancer cell proliferation and survival and potentiating the effect of chemotherapy, miR-34a exhibits low toxicity to normal cells *in vitro* and *in vivo*.^6, 15, 21, 90^ Importantly, miR-34a modulation was shown to affect to some extent miR-34a targets in the context of disrupted p53 function.

Unexpectedly, clinical trials of solid cancer treatment with miR-34a mimic delivered by liposomal nanoparticles noted severe adverse effects of immune character in five patients. These effects may be related to miR-34a-specific modulation of gene expression, but could also originate from a reaction to the liposome-based carrier or delivered double-stranded RNA molecules.^108^ Different delivery approach or improved dosing schedule, addressing the issues of cellular uptake and *in vivo* stability, could improve the safety and tolerability of miR-34a application and create an opportunity of combination therapy.

Despite targeting miR-34a containing liposomes to tumor tissues, an effect on tumor-associated immune and stromal cells and their function cannot be avoided.^85, 109^ Apart from tumor tissue, miR-34a was shown to adversely affect age- and myocardial infarction-associated viability and senescence of cardiomyocytes^110^ and processes associated with pulmonary fibrosis.^111^ Investigation of a particular miR-34a ‘targetome’ in different cell types may justify appropriately targeted usage of miR-34a mimics in cancer therapy.

With the perspective of therapeutic miR-34a introduction or re-expression, the existence of multiple feedback regulatory mechanisms of miR-34a urges considering the mechanisms, which may affect both downstream miR-34a targets and upstream miR-34a regulators. On the background of disrupted p53 function encountered in a high proportion of tumors, alternative p53-independent mechanisms of miR-34a regulation merit special attention. Mechanisms inhibiting therapeutic re-expression of miR-34a in the context of EMT, inflammation or oncogene signaling (Figure 2) could be responsible for insufficient therapeutic effect, while aberrant expression of miR-34a in normal cells or massive necrotic cell death observed in miR-34a treated tumors^21^ may underlie systemic negative effects of therapy. Successful management of side effects on non-tumor cells is indispensable for successful therapeutic application.

## Publisher’s Note

Springer Nature remains neutral with regard to jurisdictional claims in published maps and institutional affiliations.

## Figures and Tables

**Table 1:** Outcomes of miR-34a modulation in models bearing p53 mutation

**Cancer type**	**Target cells**	**Type of p53 defect**	**Type of miR-34a modulation**	**Outcome**	Reference
Glioblastoma	U251	R273H	Introduction of miRNA mimic	Inhibition of cell growth, cell cycle arrest, induction of apoptosis, reduction of *in vitro* migration and invasion capabilities	18
Myeloid leukemia	SKMM1	R248H	Introduction of synthetic miRNA oligos, stable overexpression	Growth inhibition	21
	RPMI-8226	E285K	Introduction of synthetic miRNA oligos; intratumoral injection of formulated miR-34a	Growth inhibition; anti-tumor effect	21
	OPM1	5 mutations in exons	Introduction of synthetic miRNA oligos	Growth inhibition	21
	KASUMI-1	R248Q	Introduction of miRNA precursors	Reduction of PD-L1 expression	31
Lung cancer	SBC-5	R248L	Introduction of miRNA precursor	Inhibition of proliferation, sensitization to cisplatin-based therapy	44
	H23	M246I	Introduction of synthetic miRNA oligos	Potentiation of the effect of EGFR inhibition	104
	Calu-6	R196Stop	Introduction of synthetic miRNA oligos	Potentiation of the effect of EGFR inhibition	104
	PC-9	R248Q	Introduction of synthetic miRNA oligos	Restoration of sensitivity to gefitinib	105
	344SQ	R172HΔG	Liposomal *in vivo* delivery of mature miRNA	Downregulation of immune-suppressive PDL-1, prevention of immune evasion	48
Breast cancer	MDA-MB-231	R280K	Inducible overexpression	Inhibition of cell migration and invasion	76
	BT-549	R249S	Introduction of miRNA precursor	Inhibition of cell migration and invasion	32
Ovarian cancer	SK-OV-3	H179R	Introduction of miRNA mimic	Reduced proliferation, motility, and invasion, no induction of apoptosis	20
	SK-OV-3	H179R	Introduction of miRNA mimic	Restoration of epithelial phenotype, downregulation of Snail	90
	ES-2	S241F	Introduction of miRNA mimic	Restoration of epithelial phenotype, downregulation of Snail	90
Prostate cancer	DU145	P223L V274F	Introduction of synthetic miRNA oligos	Inhibition of cell proliferation and clonal expansion	47
	DU145	P223L V274F	*In vivo* co-delivery of doxorubicin and miR-34a using polypeptide-based cationic micelles	Inhibition of xenograft growth, induction of apoptosis/necrosis *in vitro*	112
	DU145 - taxane resistant	P223L V274F	Rubone-induced stabilization combined with paclitaxel	Decreased cell viability	113
Bladder cancer	5637	R280T	Introduction of miRNA precursor	Reduced clonogenic capacity, induction of senescence, sensitization to cisplatin	45
	T24	In-frame deletion of Y126	Introduction of miRNA precursor	Reduced clonogenic capacity, induction of senescence, sensitization to cisplatin	45
Colon cancer	SW480	R273H P309S	Inducible overexpression	Cell cycle arrest, downregulation of cell cycle regulators	42
	SW480	R273H P309S	Inhibition by antagomir	Induction of EMT-driving transcription factor Snail	12
	SW480	R273H P309S	Inducible overexpression of pri-miR-34a	Restoration of epithelial phenotype, downregulation of stemness markers	12
	SW480	R273H P309S	Introduction of miRNA mimic; inducible overexpression	No change in p53 expression	38, 42
Liver cancer	HuH7	Y220C	Systemic liposomal delivery in mice	Decreased orthotopic tumor mass	114

**Table 2:** Outcomes of miR-34a modulation in models lacking p53 expression due to genomic deletion

**Cancer type**	**Target cells**	**Type of miR-34a modulation**	**Outcome**	Reference
Lung cancer	H1299	Inducible overexpression	Inhibition of migration and invasion	76
	H1299	Transfection of expression plasmid	Inhibition of colony formation, induction of apoptosis	15
	H1299	Introduction of miRNA precursor	Suppressed DNA repair after irradiation, sensitization to radiotherapy in xenograft tumors	115
	H1299	Liposomal *in vivo* delivery of mature miRNA	Downregulation of immune-suppressive PDL-1, prevention of immune evasion	48
	H358	Introduction of synthetic miRNA oligos	Potentiation of the effect of EGFR inhibition	104
Breast cancer	4T1	Introduction of miRNA precursor	Decreased tumor growth and Treg recruitment	86
Prostate cancer	PC3	Introduction of miRNA precursor	Cell growth inhibition, chemosensitization to camptothecin	116
	PC3	Introduction of synthetic miRNA oligos	Inhibition of cell proliferation, induction of apoptosis	47
	PC3 - taxane resistant	Rubone-induced stabilization combined with paclitaxel	Inhibition of tumor growth, decreased cell viability	113
Gastric cancer	Kato-III	Introduction of miRNA mimics	Growth arrest, chemosensitization and apoptosis	117
Myeloid leukemia	HL-60	Introduction of miRNA precursors/inhibitor	Reduction/restoration of PD-L1 expression and PD-L1 specific T cell apoptosis	31
