# Correction Alternative mechanisms of miR-34a regulation in cancer

**DOI:** 10.1038/s41419-018-0833-1

**Published:** 2018-07-16

**Authors:** Eva Slabáková, Zoran Culig, Ján Remšík, Karel Souček

**Affiliations:** 1 Department of Cytokinetics, Institute of Biophysics of the Czech Academy of Sciences, Brno, Czech Republic; 2 Center of Biomolecular and Cellular Engineering, International Clinical Research Center, St. Anne’s University Hospital Brno, Brno, Czech Republic; 3 Division of Experimental Urology, Department of Urology, Medical University of Innsbruck, Innsbruck, Austria; 4 Department of Experimental Biology, Faculty of Science, Masaryk University, Brno, Czech Republic

Correction to: *Cell Death & Disease*; 10.1038/cddis.2017.495; published online 12 Oct 2017.

The PDF and HTML versions of the article have been updated to include the Creative Commons Attribution 4.0 International License information.

